# A Critical juncture in global health: Leveraging historical institutionalism to examine PEPFAR dependency and inform the development of self-reliant public health systems

**DOI:** 10.1371/journal.pgph.0004440

**Published:** 2025-04-28

**Authors:** Alison Wiyeh, Patience Komba, Samuel Akombeng Ojong, Charles S. Wiysonge, Bih Moki-Suh, Patricia Sadate-Ngatchou, Ferdinand C. Mukumbang

**Affiliations:** 1 Department of Epidemiology, University of Washington School of Public Health, Seattle, Washington, United States of America; 2 Department of Global Health, University of Washington School of Public Health, Seattle, Washington, United States of America; 3 Women’s and Reproductive Health Concentration, Johns Hopkins Bloomberg School of Public Health, Baltimore, Maryland, United States of America; 4 Cochrane South Africa, South African Medical Research Council, Cape Town, South Africa; 5 PNP Ahead Limited Liability Company, Sammamish, Washington, United States of America; McGill University, CANADA

## Abstract

The U.S. President’s Emergency Plan for AIDS Relief (PEPFAR) has played an important role in expanding access to antiretroviral therapy (ART) and significantly reducing HIV/AIDS mortality globally. However, policy shifts in the United States(US) aimed at realigning foreign aid with US national interests have introduced significant uncertainty regarding PEPFAR funding in 2025, threatening to undermine decades of progress in the global HIV/AIDS response. Many countries that have long relied on PEPFAR funding are trapped in a cycle where sustained donor aid has limited their transition to self-reliant, country-led HIV programs. We leverage historical institutionalism to examine how past structures, especially colonial-era institutions, have constrained African nations and limited their capacity for self-determination through the phenomenon of path dependency. Foreign aid, often aligned with the geopolitical and economic interests of donor nations, has further undermined institutional resilience in aid-recipient countries. The recent halt in PEPFAR funding, marks a critical juncture in global health, with the potential to catalyze long-overdue systemic reforms in African health systems. As uncertainty around U.S. foreign aid grows, we anticipate an increase in engagement from private and philanthropic funders. Without corrective action, the persistence of inefficient institutional pathways will continue to undermine the impact of financial investments in many African institutions, jeopardizing the effectiveness and sustainability of initiatives aimed at improving national health outcomes. For sustainable reforms in former colonies, countries must first acknowledge the constraints of colonial legacies, decolonize mindsets and institutions, define their own development priorities, and establish transparent, accountable governance, alongside political stability as fundamental pillars for progress. PEPFAR is the first major global health program to be affected, and it is unlikely to be the last. How much longer will recipient countries remain dependent on donor funding to safeguard the health and lives of their populations?

## Introduction

In her prophetic 2009 book titled “*Dead Aid: Why Aid Is Not Working for Africa*,” Dambisa Moyo asked a question that, at the time, seemed hypothetical, even distant: *“What if, one by one, African countries each received a phone call... telling them that in exactly five years the aid taps would be shut off – permanently*?”[1] Even more concerning is that the lifeline of HIV treatment aid is being cut off abruptly, without the five-year notice that Moyo had envisioned.[2,3]

The United States President’s Emergency Plan for AIDS Relief (PEPFAR) is an unparalleled commitment to global health and remains the largest investment by any nation to combat a single disease.[4] Before PEPFAR was launched in 2003 as an emergency response to the HIV/AIDS crisis, the pandemic had already claimed over 20 million lives, with nearly 5 million new infections occurring each year, placing immense strain on health systems worldwide.[4,5] As of 2025, PEPFAR’s investments in antiretroviral therapy (ART) and other life-saving interventions of over 120 billion USD[6] is estimated to have saved more than 26 million lives, prevented HIV transmission to 7.8 million infants, and protected over 8 million children from orphanhood.[4,7] It is worth noting that nearly a quarter of countries worldwide receive PEPFAR funding, with African nations accounting for 49% (27 out of 55) of all PEPFAR-supported countries.[8] Also, PEPFAR not only funds HIV programs in recipient countries but also creates jobs for American organizations, including companies that help to expand ARTs access, as well as academic institutions that receive research, education, and implementation funding.[9–11] Historically, over 70% of the Global Health Bureau’s funding for programs like PEPFAR has been spent on overhead costs for U.S.-based non-governmental organizations(NGOs).[12] Beyond its impact on HIV/AIDS, PEPFAR has established laboratory networks, diagnostic platforms, and supply chain systems leveraged for broader disease response and public health emergencies.[13,14] For example, during the COVID-19 pandemic, PEPFAR supported diagnostic and surveillance platforms, enhanced testing, contact tracing, and outbreak response in low and middle-income countries (LMICs).[13,15,16]

Established under a Republican administration, PEPFAR has historically benefited from strong bipartisan support and broad political backing.[17] However, the difficulties in securing a five-year reauthorization in 2024 signaled a shift in the policy landscape, shaped in particular by an increase in mistrust, political polarization and misinformation.[17] On January 20, 2025, President Donald J. Trump began his second term as the 47th President of the United States and issued an executive order imposing a 90-day pause on new obligations and disbursements of U.S. foreign development assistance to evaluate program efficiency and alignment with U.S. foreign policy, along with a review of foreign assistance programs.[18] In an unprecedented move, the U.S. State Department issued a “stop-work order”, on January 24^th^ 2025, effectively freezing all funding to PEPFAR, including existing grants and contracts.[2,3,19,20] On February 1st, 2025, a temporary waiver was issued on certain aspects of the funding freeze.[21] Nonetheless, the long-term sustainability of PEPFAR remains uncertain.[19]

Since its creation in 2003, there have been growing calls for PEPFAR-supported countries to reduce donor reliance by strengthening domestic health systems and implementing sustainable healthcare financing.[22] For example, the 2008 Tom Lantos and Henry J. Hyde United States Global Leadership Against HIV/AIDS, Tuberculosis, and Malaria Reauthorization Act called for countries to strive towards country ownership of their programs.[23,24] However, many PEPFAR-supported countries continue to face structural and financial challenges that perpetuate a cycle of dependency on external assistance.[25]

One might question why, after 21 years of PEPFAR support and over $120 billion in investment, PEPFAR-funded countries remain reliant on external assistance, lacking a clear framework for sustainable, country-led HIV/AIDS responses. The causes are undoubtedly multifaceted. We contribute to the ongoing discourse by asserting that historical institutions have played a critical role and must be confronted not as remnants of the past but as persistent forces that continue to shape present-day systems and policies through path dependency. Path dependency is a phenomenon in which early decisions influence and constrain long-term institutional trajectories, making change difficult.[26] To achieve sustainable progress, we must recognize and actively dissolve these path dependencies, establishing new institutions grounded in national priorities so as to foster self-driven development.[27]

## Historical institutions: The enduring chains that have kept Africa bound

Institutions are the deep-rooted unseen forces shaping how our world works. They include rules, norms, traditions, and systems passed down over time.[28,29] Institutions serve as roadmaps defining what is possible, what is acceptable, and what is expected, influencing everything from governments to economies and healthcare systems.[30] Historical institutionalists argue that current institutions are deeply shaped by historical trajectories, reflecting the legacies of pre-colonial, colonial rule, and postcolonial governance.[26,31–34]

In many African countries, the pre-colonial period was characterized by indigenous governance systems, economic structures, and social organizations deeply rooted in local customs, traditions, and values.[35] Political institutions varied widely, from centralized kingdoms and empires like the Shongai in Western Africa [33,36] to decentralized, stateless societies like the Nuer in Sudan.[36] Many African societies had well-established governance structures that included checks on power, communal resource management, and dispute-resolution mechanisms.[35]

Then came the colonial era, which saw European powers expand their territorial control across Africa, with large-scale colonization peaking in the late 19th and early 20th centuries.[37] While colonial experiences varied across African nations and colonizers, the institutions established shared a common objective: to advance colonial interests and enforce systemic subjugation. [26,38] The duration of colonial rule spanned different lengths, with some countries, such as Mozambique, undergoing a progressive and evolving colonization process that spanned over 470 years, deeply affecting the lives of more than 15 generations under foreign control.[39] There were significant institutional disruptions in Africa as European powers sought to restructure governance, economic, and social systems to align with imperial interests.[40] In settler colonies, such as South Africa, long-term governance structures and institutional frameworks were established to support European land ownership and settlement.[41] In contrast, in extractive colonies like the Belgian Congo, resource exploitation was prioritized with minimal investment in governance or local institutional development.[33]

Colonial administrations established centralized bureaucracies prioritizing administrative control over participatory governance and local institutional continuity.[41,42] While British indirect rule leveraged existing local institutions to administer colonial governance, the French pursued a policy of assimilation and centralized control, often replacing or restructuring indigenous systems to align with French administrative and cultural models.[43] Colonial boundaries were drawn without regard for ethnic, linguistic, or cultural divisions, resulting in a split of cohesive groups across multiple territories while forcing unrelated groups into single political entities.[44] Colonial administrators centralized power in appointed intermediaries who served colonial interests over local governance, undermining transparency and dismantling existing accountability structures.[41] They used divide-and-rule strategies, selectively favoring certain ethnic groups and regional elites to maintain control. This approach institutionalized patronage, eroding merit-based governance, exacerbating inequalities in resource distribution, and normalizing leadership as a conduit for personal gain. [42,45] African collaboration was the backbone of European colonialism, serving as the crucial link between colonial rulers and the governed populations. Chiefs, teachers, soldiers, and interpreters—willing and reluctant—became the hands and voices of imperial power. Some sought survival, others advantage, drawn by the promise of wealth, influence, or status. Nevertheless, through them, colonial rule was upheld.[46]

Colonial powers implemented differing education policies with lasting impacts. For example, in French colonies, education was highly centralized and assimilationist, with limited access for Africans. Those who did receive schooling were often alienated from their native cultures, contributing to high illiteracy rates.[43] By the late 1960s, up to 95% of the population in France’s former Black African territories remained illiterate.[47]

Colonial economic policies equally varied, shaping long-term development. French colonies enforced protectionist trade systems, restricting commerce to the metropole and requiring strict political control.[43] Their policies often mandated that colonies export raw materials exclusively to the metropole while importing finished goods from their colonial rulers, creating economic dependency and stifling industrial growth. Additionally, the strict political control imposed by these powers ensured that colonial economies remained tightly integrated with and subordinate to the economic needs of France.[43]

The historical development of healthcare systems in colonized regions was not structured to ensure equitable access to medical services for local populations.[48] Rather, colonial medical frameworks were primarily established to safeguard the health of colonial administrators, military personnel, and labor forces deemed essential for resource extraction and economic productivity.[48–50] As a result, healthcare infrastructure, workforce training, medical research, and policy initiatives were designed to serve colonial administrative priorities rather than to build independent, resilient health systems focused on equitable healthcare access.[50]

Although formal colonial rule in Africa has ended, many African nations continue to experience the enduring effects of colonialism due to historical legacies and existing systems.[43,51,52] For example, colonial rule systematically devalued African cultures, imposing foreign values and systems that led many Africans to internalize feelings of inferiority, reinforcing a sense of subordination that has persisted over many generations till today.[53] After independence, many African nations retained centralized governance structures as well as ethnic and elite-based power structures, enabling corruption and authoritarian rule. This has contributed to political instability, weak national unity, and governance crises, reinforcing path dependency where new political structures cannot easily emerge.[54,55] The education systems in many African countries have remained largely unchanged since colonization, continuing to mirror colonial patterns rather than evolving to train students in entrepreneurship or industrialization for economic diversification and transformation.[56] Similarly, colonial health structures have persisted, further exacerbated by underdevelopment in key sectors such as education, governance, and economic stability. Health systems do not function in isolation; their effectiveness depends on the strength of interconnected systems. In the following section, we examine how attempts to transition away from these inherited institutions have been significantly constrained by path dependency.[57]

## Path dependency and PEPFAR: Implications for African HIV programs

Path dependency, a concept rooted in historical and institutional economics, describes how past decisions and institutional legacies establish self-reinforcing mechanisms that limit future policy options.[58,59] Once an institution or organization follows a certain path, its policies, systems, and investments build on each other, making it harder and more costly to change direction over time.[26] Political, social, and professional structures incentivize policymakers to maintain existing systems, as reforms could disrupt vested interests, institutional stability, and established alliances. [60,61] As a result, transitioning from an entrenched system incurs high financial and administrative costs, demanding substantial investment, policy restructuring, and social adaptation, making reforms complex and politically sensitive.[62]

**Fig 1:**
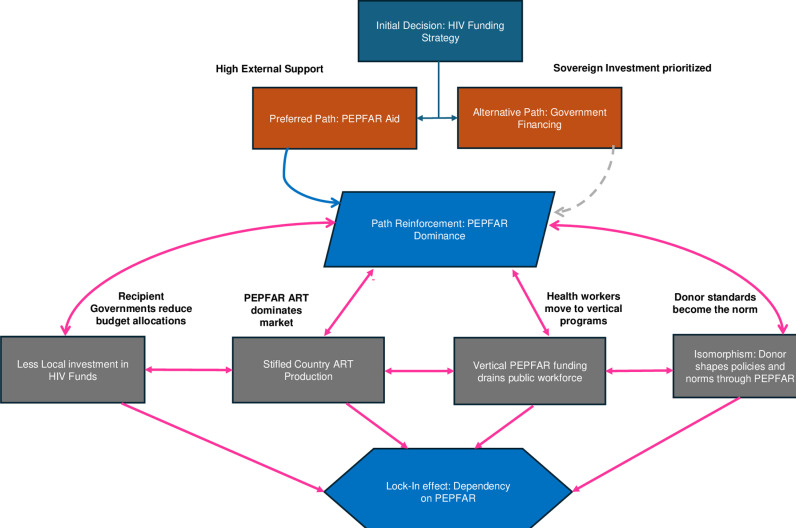
Path Dependency in HIV Funding.

Aid through the Marshall Plan (European Recovery Program) was instrumental in rebuilding Europe’s political and social structures after World War II.[63,64] In contrast, aid to Africa has largely reinforced path dependency, perpetuating poverty and governance challenges.[1] PEPFAR exemplifies how external funding dominates HIV/AIDS programs in some African countries, covering over 95% of services. This heavy reliance creates structurally fragile health programs that remain dependent on external support, limiting their ability to function independently, as shown in Fig 1.[65] Its vertical, disease-specific approach has further fragmented healthcare delivery, preventing HIV/AIDS programs from fully integrating into national health infrastructures.[66]

The program has also disrupted health workforce distribution. Offering higher salaries for HIV/AIDS specialists has created a shift in workforce allocation, drawing professionals away from other critical health areas such as maternal health and non-communicable diseases. This has weakened primary healthcare delivery systems and the overall health system.[67] Similarly, its centralized procurement system for ARTs has left recipient countries struggling to develop independent supply chains. A key example is the importation of foreign-made ARVs, which, while expanding treatment access, leads to reliance on external manufacturers, limiting the growth of domestic manufacturing capabilities.[10,68] This has led to what Moyo calls the micro-macro paradox, where aid succeeds at the project level but fails to drive broader economic growth.[1] As PEPFAR-controlled procurement phases out, many nations face higher costs and logistical challenges, making drug supply sustainability uncertain.[67] Another consequence of PEPFAR’s approach is the erosion of domestic health investments. Because PEPFAR tends to cover most HIV/AIDS costs, governments deprioritized local funding for HIV care, reinforcing financial dependency.[25]

With the U.S. signaling potential funding cuts, PEPFAR-reliant countries face an imminent crisis. Since the programs did not establish financial or logistical self-sufficiency mechanisms, a sudden withdrawal could disrupt ARTs for millions, increasing viral resistance, opportunistic infections, and mortality.[67,19] A survey of PEPFAR beneficiaries across 27 countries in the week following the funding freeze revealed immediate disruptions in HIV programs.[69] Among the 153 respondents, 70% reported that their organizations had to cancel HIV services, with over 90% stopping or reducing patient follow-up, support for survivors of gender-based violence, data tracking, HIV testing, and HIV treatment each.[69] Only 14% of respondents reported that their organizations could sustain operations for more than a month without PEPFAR support.[69] Further, a model-based analysis suggests that with full PEPFAR funding, 1.19 million new HIV infections are expected over 10 years, and people with HIV would have a life expectancy of 61.4 years.[70] In hypothetical scenarios where PEPFAR funding was reduced to 50% or eliminated, an additional 286,000 and 565,000 infections occurred, respectively, with life expectancy decreasing by 2 to 3.7 years.[70] The full impact will only become evident after months to years of systematic tracking and assessment.[71]

## The Trump-Era restrictions: A Critical juncture for change?

Once a path dependence has been established, institutions typically experience prolonged periods of stability, occasionally disrupted by brief phases of significant change, known as Critical Junctures. These junctures occur when external shocks, policy shifts, or crises disrupt existing institutional trajectories, creating windows of opportunity for significant reform.[32] At these critical junctures, decision-makers have a broader range of options where their choices have a profound and lasting impact on institutional trajectories.[72,73] Decisions made in these moments set institutions on new paths, reinforcing patterns of stability until another critical juncture emerges, perpetuating the cycle of path dependence.[74,75] We believe that the recent restrictions on foreign aid for global health under the Trump administration represent a critical juncture, offering an opportunity for recipient countries to strengthen health sovereignty and take greater ownership of their health responses.[76]

Critics contend that when countries are entrenched in path dependency, they often lack the fiscal capacity and institutional frameworks needed to transition toward self-sufficiency, making external disruptions necessary for change.[62] However, evidence suggests that breaking away from path dependency is achievable.[34] Moreover, institutional change is not limited to critical junctures but can also unfold gradually over time through adaptive evolution.[27] Research identifies five key modes of gradual yet transformative change—displacement, layering, drift, conversion, and exhaustion—as summarized in Table 1.[27]

**Table 1:** Types of institutional change (Adapted from Streeck & Thelen [27]).

Type	Description	Key Mechanism	Process of Change
Displacement	The gradual rise of previously marginalized institutions or ideas, replacing dominant ones over time.	Defection	Actors actively promote an alternative institutional logic, leading to a shift in authority.
Layering	The introduction of new policies or structures alongside existing ones, altering their function without direct replacement.	Differential Growth	Emerging institutions expand at a faster rate than older ones, progressively reducing their dominance.
Drift	Institutions remain formally unchanged but gradually lose effectiveness due to shifts in their external environment.	Deliberate Neglect	Failure to adapt policies or enforce regulations results in unintended shifts in institutional outcomes.
Conversion	Existing institutions are repurposed to serve new objectives, even while their formal structure remains intact.	Redirection & Reinterpretation	Institutional actors reinterpret existing rules and practices to align with evolving needs.
Exhaustion	Institutions experience gradual decline as their internal dynamics erode their effectiveness over time.	Depletion & Overuse	Diminishing returns, overextension, or changing cost-benefit dynamics weaken institutional stability.

We recognize that historical institutions have placed African countries on distinct path dependencies and the solutions are complex.[77] Addressing path dependency requires recognizing the continued constraints on former colonies, decolonizing mindsets, dismantling colonial legacies, fostering transparent governance, political stability, and intentional commitment to institutional reform as pillars for success.[1] Time-bound interventions that strengthen domestic financing through innovative revenue generation and diversification strategies, support economic restructuring for sustainable growth, and systematically reduce dependence on donor funding will be crucial.[1] African nations should prioritize loco-regional procurement and manufacturing strategies, complemented by structured knowledge exchange with countries that have successfully mitigated similar path dependencies. With growing uncertainty around PEPFAR funding, private sector investment in health is likely to expand; however, without robust governance and strategic preparedness, both aid and private sector partnerships risk inefficiency and systemic instability. Furthermore, while we operate within the public health sector, we acknowledge that the sustainability and effectiveness of this sector is deeply interconnected with developments in other sectors, including governance, education, infrastructure, and economic sectors.[78] Hence, a holistic, cross-sectoral approach is essential to achieving meaningful and lasting reform.[79]

African nations can use this crisis to drive essential reforms in HIV and other public health programs, drawing on lessons from peers successfully navigating similar transitions for guidance.[80] For example, evidence from South Africa’s National Strategic Plan (NSP) for HIV shows that countries making intentional financial and policy shifts toward domestic ownership can reduce their reliance on external aid.[80] For instance, between 2017 and 2020, South Africa’s domestic contribution to HIV funding ranged from 69% to 77%, demonstrating a strong commitment to sustaining its HIV response.[81] As a result, South Africa provides ART to 5.6 million people, funding medication procurement, while PEPFAR continues to support staffing, prevention, medical circumcision, and public health messaging.[65] Botswana’s HIV Sustainability and Transition Roadmap outlines strategies to mitigate risks from reduced donor support, emphasizing increased domestic funding, strengthened healthcare systems, and private sector partnerships. These efforts have enabled Botswana to sustain its HIV response with reduced reliance on PEPFAR.[82,83] Both countries’ experiences underscore the importance of strategic planning, capacity building, and domestic investment in achieving sustainable HIV program management post-PEPFAR. The key question is whether these forced transitions will drive positive, long-term improvements in health system resilience or instead create new vulnerabilities that further destabilize public health infrastructure.

In the interim, governments must allocate emergency funds to sustain essential HIV services while actively seeking to diversify donor support. Service delivery models must be adapted to reduce dependence on centralized clinics, ensuring uninterrupted access to care. Accelerating local and regional production of generic antiretroviral drugs is also crucial to minimizing reliance on external donors and preventing stockouts. Ultimately, these efforts should be directed toward achieving full national ownership of HIV programs, ensuring long-term sustainability and resilience

If the U.S. is serious about redefining PEPFAR’s role, it must prioritize a responsible transition, not an abrupt withdrawal. A gradual funding phase-out, paired with strategic local investments, would allow countries to assume greater financial responsibility.[84] A genuine commitment to global health justice requires funding programs and ensuring that they empower countries to sustain them long after donors leave. Without such foresight, PEPFAR risks becoming another example of how well-intentioned interventions can reinforce, rather than dismantle, colonial-era dependencies.

History has shown that no system is too entrenched to be reformed, no structure too rigid to be dismantled. The time to reclaim ownership of health financing is now, before another funding crisis exposes the fragility of an age-old aid-dependent system.
